# Opposing roles of the dorsolateral and dorsomedial striatum in the acquisition of skilled action sequencing in rats

**DOI:** 10.1523/JNEUROSCI.1907-21.2022

**Published:** 2022-01-27

**Authors:** Karly M. Turner, Anna Svegborn, Mia Langguth, Colin McKenzie, Trevor W. Robbins

**Affiliations:** 1 Department of Psychology, University of Cambridge, Cambridge CB23EB, United Kingdom; 2 Behavioural and Cognitive Neuroscience Institute, University of Cambridge, Cambridge CB23EB, United Kingdom; 3 School of Psychology, University of New South Wales, Sydney 2052, Australia

## Abstract

The shift in control from dorsomedial to dorsolateral striatum during skill and habit formation has been well established, but whether striatal subregions orchestrate this shift co-operatively or competitively remains unclear. Cortical inputs have also been implicated in the shift towards automaticity, but it is unknown if they mirror their downstream striatal targets across this transition. We addressed these questions using a five-step heterogeneous action sequencing task in male rats that is optimally performed by automated chains of actions. By optimising automatic habitual responding, we discovered that loss of function in the dorsomedial striatum accelerated sequence acquisition. In contrast, loss of function in the dorsolateral striatum impeded acquisition of sequencing, demonstrating functional opposition within the striatum. Unexpectedly the medial prefrontal cortex was not involved, however the lateral orbitofrontal cortex was critical. These results shift current theories about striatal control of behavior to a model of competitive opposition, where the dorsomedial striatum interferes with the development of dorsolateral-striatum dependent behavior.

## Introduction

There are mixed views on exactly how habits and skills interact. They are two separate descriptors of behavior with overlapping and distinct features (Dezfouli and Balleine, 2012; Graybiel and Grafton, 2015; Jin and Costa, 2015; Robbins and Costa, 2017). Skills describe refined behavioral repertoires, which may be under goal-directed or habitual control (or a combination). In contrast, habits are defined as responses that are triggered by stimuli and are autonomous of the outcome value but may include skilled and unskilled behaviors. The concept of automaticity - where behavior becomes stereotypical, performed with little variation in a highly efficient manner and without effortful thought - captures many of the shared elements between habits and skills (Ashby et al., 2010). Chunked action sequences provide an opportunity to study the nexus of automaticity, skills, and habits (Dezfouli et al., 2014; Graybiel and Grafton, 2015; Robbins and Costa, 2017). The development of automaticity in both habits and skills is paralleled by a well-documented shift in control from the dorsomedial (DMS) to dorsolateral (DLS) striatum (Yin et al., 2009; Ashby et al., 2010; Thorn et al., 2010; Graybiel and Grafton, 2015; Kupferschmidt et al., 2017). Yet, it is unknown how this transition occurs and how these regions co-ordinate the control of actions.

Goal-directed behavior, dependent on DMS function, dominates early in instrumental conditioning but if conditions support habitual responding then the DLS takes control (Yin et al., 2004, 2005a; Yin et al., 2005b; Yin and Knowlton, 2006; Yin et al., 2006; Balleine et al., 2009). Similarly, in skill learning there is an early learning phase where actions are variable and slow but control shifts to the DLS as they become refined and efficient (Miyachi et al., 2002; Lehericy et al., 2005; Yin et al., 2009; Kupferschmidt et al., 2017). Neural studies indicate the DMS and DLS operate in parallel during this transition with some degree of interdependency (Yin et al., 2009; Vandaele et al., 2019). Recently it has been shown that the DLS is engaged from the beginning and only after initial experience does the goal-directed system start driving behavior (Kupferschmidt et al., 2017; Bergstrom et al., 2018; Smith et al., 2021). It is unclear whether they act via a co-operative or competitive relationship (Balleine et al., 2009; Smith and Graybiel, 2016b). Dual control accounts suggest the two behavioral processes both contribute with the relative influence shifting with extended training (Dickinson, 1985; Robbins and Costa, 2017; Balleine, 2019; Balleine and Dezfouli, 2019), possibly reflecting the summation of goal-directed and habitual processes (Perez and Dickinson, 2020). Similar accounts may apply to the relative neural contribution of the DMS and DLS to action control.

If these regions operate independently, then loss of function should impair only that region’s function, however if they operate co-operatively then both functions would be impaired (e.g. loss of DMS also impairs habits). In contrast, an opponent relationship would predict that loss of function in one region would favour the alternate structure’s function (e.g., loss of DMS *enhances* habits). A problematic issue when addressing this question has been the “zero-sum” interpretation as habits are defined by a lack of goal-directed features (Robbins and Costa, 2017; Schreiner et al., 2020). A lack of devaluation sensitivity may result from impaired instrumental learning, rather than habits (Balleine and Dezfouli, 2019). In addition, habits are typically identified by an impairment in action modification when conditions change (e.g., devaluation), but rarely as the optimal response. Hence, we developed a novel rodent paradigm using a sequence of heterogeneous actions where automated, reflexive responding would lead to superior performance, to test models of striatal control during the development of automaticity. We hypothesised that DMS loss of function would causally accelerate, whereas DLS loss of function would impair, the development of behavioral automaticity.

## Materials and Methods

The task was developed in treatment naïve rats where we examined the effects of extended training and then the inclusion of punishment for incorrect sequences. Using this refined protocol, we then conducted three experiments in separate cohorts of rats examining the effect of pre-training lesions of the (1) DMS and DLS; (2) mOFC and lOFC; and (3) PrL and IL on acquisition of action sequencing. Methods were the same across these experiments, with exceptions detailed below.

### Animals and Housing

Adult male Lister-hooded rats weighing 280-300g (Charles River, UK) were housed in groups of four on reversed 12-h light cycle (off at 07:00) within a temperature (21°C) and humidity-controlled environment in open top cages with aspen bedding, wood block and tube. A week after arriving, rats were food-restricted to no less than 90% of free-feeding weight with unrestricted access to water and were exposed to reward pellets. All procedures were conducted in accordance with the United Kingdom Animal (Scientific Procedures) Act of 1986 and were approved by ethical review at the University of Cambridge.

### Apparatus

Rats were trained to perform a five-step sequential nose poke task (SNT), which was adapted from Keeler et al. (2014), however with substantial changes including absence of cues and the number and order of responses. The task was conducted in operant chambers (Campden Instruments, UK) with five nose poke apertures available within a horizontal array and a reward receptacle on the opposing wall (Robbins, 2002). Nose pokes and the reward receptacle were fitted with infra-red beams to detect head entries and a light for illumination. Reward sucrose pellets (AIN76A, 45mg; TestDiet, UK) were delivered into the receptacle by a pellet dispenser. A house light was mounted on the ceiling and the chamber was contained within a sound attenuating box. Overhead cameras (SpyCameraCCTV, UK) were mounted above each chamber to monitor and record behavior remotely. Whisker Server software and custom programming software was used to operate the chambers and record responses (Cardinal and Aitken, 2010; Keeler et al., 2014).

### Sequential Nose poke Task (SNT) Protocol

The SNT requires rats to make a nose poke response into each of the five holes from left to right across a horizontal array to receive a food reward. Sessions ran for 30 min unless stated otherwise and all nose pokes and head entries were recorded with the duration of each nose poke calculated based on the entry and exit times (Table 1). Rats were first habituated to the chambers and retrieved rewards from the receptacle that were dispensed with each head entry until 100 were collected (stage 1). Next, rats were trained to make nose poke responses into the five-hole array (stage 2). Each hole in the five-step sequence was illuminated for 1 s before moving to the next location from left to right and finishing with reward delivery (e.g. 1-2-3-4-5-Reward), which was signalled by illumination of the receptacle. Head entry into the receptacle triggered the start of the next trial. Critically, when the rat nose poked an illuminated hole, the light and sequence counter immediately moved on to the next hole, allowing the rat to achieve reward delivery faster than if they did not nose poke. If the rat made a nose poke into an alternative hole, the illuminated hole would flash for the duration of the incorrect nose poke to draw attention to the correct location. To further encourage nose poking, the illumination duration incremented by 10% of the original delay (1 s) each trial, further delaying reward delivery if nose pokes were not made. This training protocol was implemented to reduce bias for the start or end elements (inherent to training by chaining) and rapidly produced sequencing behavior. Once rats were successfully able to complete at least 15 sequences within a session, they moved to stage 3 where the illumination sequence only advanced to the next hole, and ultimately to reward delivery, after a correct nose poke response into an illuminated hole. Criteria for stage 3 was 50 complete sequences, which was typically achieved in a single session. Stage 4 was identical to stage 3, except that now the nose poke holes were no longer illuminated. After each of the holes had been poked in order, a reward was delivered. Incorrect nose pokes were recorded, but not punished. After reaching 50 uncued sequences, they were moved to the final level (stage 5) where incorrect nose pokes were punished with a 5 s time out period signalled by the illumination of the house light. After the time out ended, the rat was required to start the sequence again from hole 1. Responses during the timeout period were recorded but did not extend the time out duration. Training on stage 5 was conducted for 15 sessions and rats began immediately after reaching training criteria. Key measures included trials initiated, correct sequences, incorrect sequences, nose poke durations at each location and total sequence duration (Table 2).

It is worth addressing how these measures translate to the quantification of habits, skills, and automaticity. Firstly, skill development can be measured by the ability to perform an action in an accurate and efficient manner via procedural learning (Jog et al., 1999). Accuracy can be observed on this task as the number of correct and incorrect sequences performed, while efficiency is globally captured in the number of trials initiated given this task is time limited. Any time wasted will result in fewer trials per session. Secondly, automaticity can be measured by an increase in speed and a reduction in variation. Speed is measured through total sequence duration and the duration of each nose poke action across the holes. Reduced variance can then be assessed by comparing the coefficient of variation (CV=mean/standard deviation; (Jin and Costa, 2010)) on response times across acquisition.

How skills and automaticity interact with habits has been the topic of many recent publications (Graybiel and Grafton, 2015; Smith and Graybiel, 2016a; Robbins and Costa, 2017; Garr and Delamater, 2019). A recent definition of habits included four elements - rapid execution, invariant topography, chunked action sequences, and insensitive to change in contingency/value (Balleine and Dezfouli, 2019). Notably speed and invariance are the defining features of automaticity. The third feature, chunking of action sequences, has strong ties to skill development (Smith and Graybiel, 2016a). Sensitivity to outcome devaluation as a measure of habit was attempted in developing this task but was not suitable due to rapid extinction. Therefore, we have reserved use of the term habit to the first set of results where outcome devaluation was incorporated and automaticity for the remainder of the paper where the full definition of habit cannot be verified.

### Task development

During task development we originally only trained to stage 4. Rats were then split into two groups (n=12) with one group continuing with daily training sessions (morning only), while the extended group moved to twice daily sessions (morning and afternoon) for 10 days. Sensitivity to outcome-specific devaluation was then tested. As this did not result in habitual action sequencing, rats were then reallocated (matched for prior training history) to either continue daily training sessions at stage 4 (flexible group) or were moved to stage 5 (invariant group) where incorrect sequences were punished for 15 sessions. Rats then underwent outcome-specific devaluation testing.

### Outcome-specific devaluation

Rats were familiarised to the grain pellets in their home cage prior to devaluation testing. Individuals were placed in empty wire-top cages with free access to 25g of either grain or sucrose pellets for 30 min before being placed into the operant chambers for a 10 min test in extinction. Rats were given two standard training sessions to recover high response rates before being tested with the alternative outcome.

### Surgery

Prior to training rats were randomly assigned to receive either sham surgery or intracranial bilateral lesions to the region of interest under 2-3% isoflurane anaesthesia with local application of bupivacaine (2mg/kg s.c. at 0.8ml/kg; Sigma) at the incision site. Fibre-sparing lesions were induced by quinolinic acid (0.09M in PBS, Sigma Aldrich, UK) or phosphate-buffered saline (PBS) sham infusions at 0.1ul/min using the co-ordinates in Table 3 relative to bregma based on Paxinos and Watson (2005). Rats were treated with Metacam (1mg/kg; Boehringer Ingelheim) pre- and post-operatively and rehoused in groups of four after lesion surgery. After at least 7 days recovery, rats were food restricted and began operant training as described above.

### Histology

Rats were transcardially perfused using 0.01M PBS with 5g/L sodium nitrite followed by 4% formaldehyde. Brains were then removed for storage in 4% formaldehyde at room temperature overnight on a shaker. They were then transferred to 30% sucrose until they sank before being rapidly frozen and cut into 60um sections on a freezing microtome (Leica). Sections were stained for NeuN to confirm lesion placement.

### NeuN protocol

Sections were washed in 0.01M PBS and then placed in primary antibody (NeuN monoclonal mouse anti-neuronal nuclear protein, Millipore MAB377, 1:2000 in 0.4% Triton X-100 in 0.01M PBS) for two hours on a rotary shaker. Sections are washed three times in 0.01M PBS over 30 min, then secondary (biotinylated anti-mouse IgG, Vector Laboratories BA-2001, at 1:200 in 0.4% Triton X-100 in 0.01M PBS) applied for 90 min. Sections were washed three times in 0.01M PBS, before applying an immunoperoxidase procedure (Vectastain ABC Kit, Vector Laboratories). Sections were washed three times in 0.01M PBS before visualising in DAB (ImmPACT DAB Peroxidase (HRP) Substrate, Vector Laboratories) and stopping reaction with cold 0.01M PBS. Sections were mounted on gelatin coated slides and dried before clearing with 100% ethanol (2 min), then 50% Ethanol/50% xylene (2 min) and 100% xylene before cover slipping with DPX mountant (Sigma). Images were captured using a NanoZoomer digital slide scanner and visualised with the NDP.view software (Hamamatsu) for histological verification of lesion placement.

## Experimental Design and Statistical Analysis

### Statistical Analysis

Rats were excluded for inaccurate or insufficient lesion placement or if they failed to perform action sequences (not reaching training criteria within 20 sessions of training). Final group sizes are reported in the figure legends for each group. Acquisition data was collected over 15 sessions and averaged across blocks of three sessions leading to five blocks to better represent acquisition rates and avoid excessively conservative correction for multiple comparisons. The first and last blocks were compared to identify change across acquisition. Sequence duration was calculated from the onset of nose poke 1 to the offset of nose poke 5, while the nose poke duration was calculated from entry to exit at each hole. The median and standard deviation for each rat on each day was calculated from individual response times. Timing data was not stored by the program for four rats in one session and therefore their times were averaged across two sessions rather than three for that block to prevent exclusion from the entire dataset. Where appropriate we applied paired t-tests, univariate or repeated measures analysis of variance ANOVA, with simple effects used in the case of significant interactions or post hoc comparisons for effects between treatment groups (SPSS v.25, IBM). Greenhouse-Geisser corrections were made if the sphericity assumption was violated and epsilon was <0.75.

## Results

### A novel five-step action sequencing task for rats

**Figure 1:**
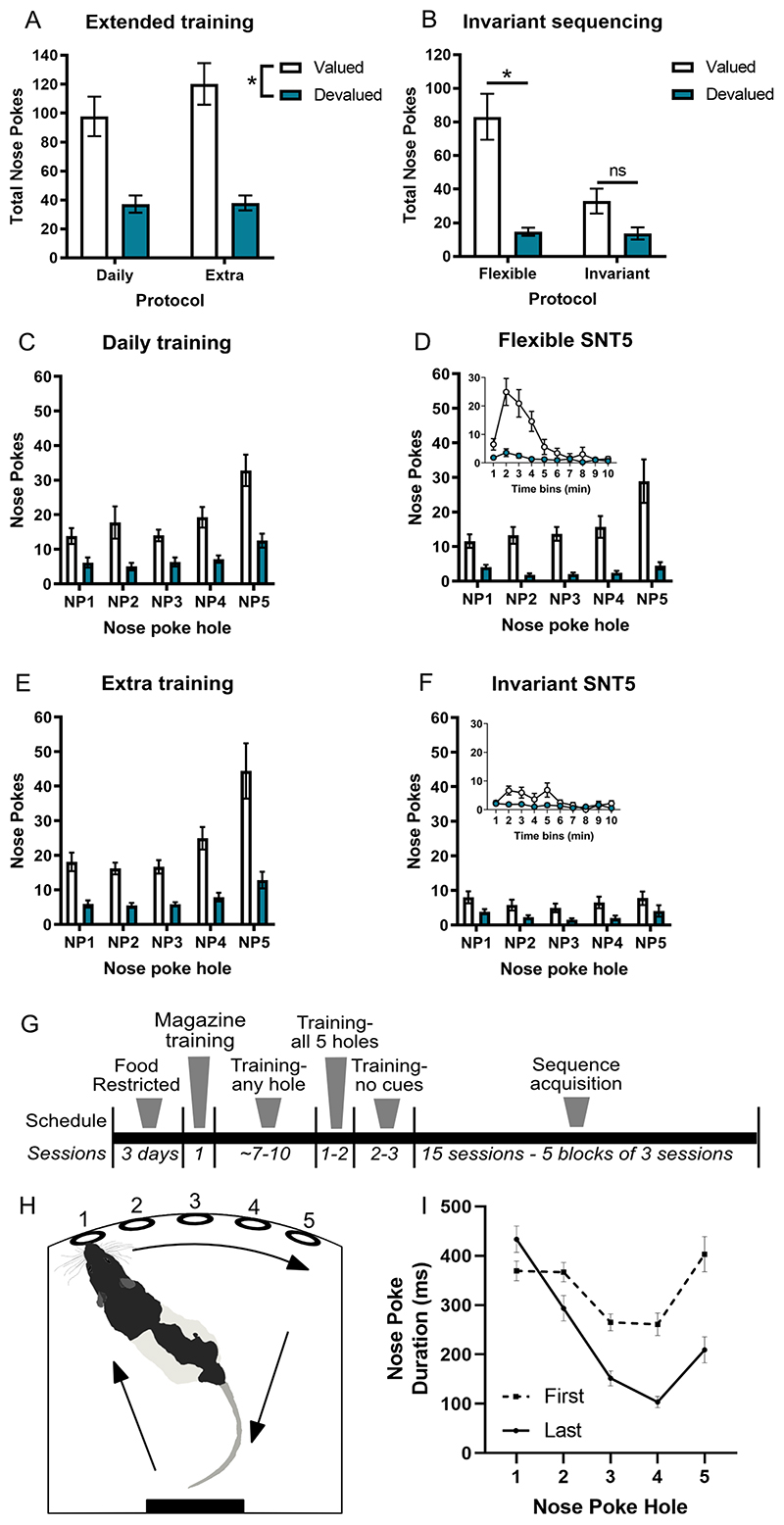
The sequential nose poke task leads to ballistic responding. (A) Extended training did not alter sensitivity to outcome-specific devaluation between the daily and extended training groups, with both groups responding more in the valued compared to devalued test session. (C) and (E) show the number of responses across the sequence elements. (B) Constraining rewards to only perfect sequences with time-outs for any errors in the invariant protocol led to habitual responding, while the flexible group remained goal-directed. (D) and (F) show the number of responses across the sequence elements under the valued and devalued conditions with insets showing total responses per minute. (G) The training schedule included habituation to the magazine and nose poke training. The nose poke cues were rapidly removed once rats were responding to each hole. From the beginning of the sequence acquisition period only correct five-step sequences were rewarded and errors were penalised by a brief time out period, after which the sequence had to be reinitiated (see Methods). (H) Rats were trained to make a five-step nose poke sequences to receive a food reward if they nose poked into each of the holes in order from left to right. (I) Rats developed a ballistic response pattern across the five holes from the first to last block of training. Each nose poke was faster and with less variance as the sequence progressed. Data shown as group mean ± S.E.M. *p<0.05.

Using a multiple-response operant chamber (Carli et al., 1983), rats made a nose poke response in each of five holes from left to right to receive a reward sucrose pellet in the magazine (Figure 1H). After brief training, rats could initiate self-paced sequences during a daily 30 min session (Figure 1G). Importantly, the sequential nose poke task was self-initiated and not cued. This required the acquisition and then retrieval of a planned motor sequence, of which the first four actions were never immediately rewarded. The removal of cues also ensured that the sequence required internal representation where enhanced performance was due to an improved representation and retrieval of the sequence rather than an improved ability to detect stimuli (Yin, 2010). It was expected that following repeated reinforcement the five individual actions would be chunked into a more efficient unitary motor program. This task would be most efficiently performed by the development of automaticity and aimed to fit the behavioral criteria for both habits and skills.

### Testing for habitual properties of the heterogenous 5-element response sequence

A classic method used to induce habitual responding is extended training (Dickinson, 1985). We trained rats to perform the sequencing task without cues and then placed half of them onto a twice daily (extended) training regime, while the other half continued with daily sessions for 10 days. Outcome-specific devaluation was then used to probe habits through sensitivity to changes in outcome value. The outcome was devalued by providing free access to 25 g of the sucrose pellets, allowing the rat to become sated, before recording sequencing responses for 10 min in extinction. This was compared to a separate counterbalanced session where rats were sated on grain pellets before testing, thereby leaving the outcome (sucrose pellets) still valued. Rats were tested in extinction to prevent learning about the change in outcome value through the experience of earning the outcome in the sated state, thereby demonstrating whether actions were influenced by changes in inferred outcome value. If the rats respond less when sated on sucrose pellets than grain pellets, then the specific value of the outcome was being used to adapt actions and the animal was responding under goal-directed control. If the rat responded equally after both the sucrose and grain pellets, then changes in outcome value were not being used to guide actions, indicative of habits. A repeated-measures ANOVA found there was no evidence of habit formation in either group with a significant effect of Devaluation (F_1,22_=67.78, p<0.001) and Hole (F_2,38_=29.66, p<0.001), but no main effect of Group (F_1,22_=0.9, p=0.4) or interactions with Group (p’s>0.3) (Figures 1A, C, E). This indicates both groups trained for either 10 or 20 sessions remained goal-directed.

Another important factor in habit formation is behavioral variation (Dickinson, 1985). If rats made a sequencing mistake (most commonly skipping a hole due to insufficient nose poke depth) the program would wait for the correct response before moving to the next hole. This allowed rats to correct their mistakes and then continue with the sequence. This resulted in variation in rewarded sequence structure (e.g., **1**-**2**-4-2-**3**-**4**-**5**-**reward**) and promotes some level of self-monitoring to detect where an error was made so it could quickly be rectified. Variation in sequence structure and attending to actions to detect errors should retard habit formation. Given there were no differences detected after extended training, the rats were again split into two groups (n=12/group, balanced for prior training) with one group moving to an invariant sequencing protocol where errors were punished, and the other group continued training on the same protocol. In the new, invariant protocol, when rats made a sequencing error the house light was illuminated for 5 s and they then needed to restart the sequence from the beginning, ensuring only perfect sequences were rewarded (e.g., **1**-**2**-4-time out-**1**-**2**-**3**-**4**-**5**-**reward**). In the final three sessions prior to devaluation, rats that remained on the flexible task on average performed 139 trials of which 40 trials were perfect sequences and 99 trials had at least one sequence alteration but were completed and rewarded. In contrast, the rats that moved to the invariant protocol produced 145 trials with 79 being perfect and rewarded, and an average of 66 incorrect trials leading to a time out. Rats were then retested for habitual responding using outcome-specific devaluation as described above. Across the five holes, there was more responding on the valued compared to devalued condition and a significant interaction with Group demonstrating devaluation sensitivity was significantly reduced when only perfect sequences were rewarded (Figures 1B, D, F; Devaluation: F_1,22_=38.57, p<0.001; Hole: F_1,30_=9.39, p=0.002; Group: F_1,22_=8.19, p=0.009; Devaluation X Group: F_1,22_=12.11, p=0.002, Devaluation X Hole X Group: F_4,88_=6.95, p<0.001; repeated measures ANOVA). A simple effects test revealed that while the flexible group remained goal-directed (p<0.001), the invariant protocol led to habitual responding as indicated by the lack of a significant difference in responding between the valued and devalued conditions (p=0.07). Given the prior extent of training (the flexible group having now received >1000 action-outcome pairings) it seems unlikely further training would have led to habit formation. There was a clear reduction in the amount of valued responding with the introduction of the invariant procedure compared to the flexible group (Figure 1B). We considered if this reduction in responding during the valued session could be due to poor goal-directed learning, but the chance of producing 5 uncued actions in the correct order without knowledge of the action-outcome association during training is highly unlikely. A reduction in valued, rather than increase in devalued responding, is commonly observed in devaluation studies (Corbit et al., 2001; Bradfield and Balleine, 2017; Hart et al., 2018a) without a differential interpretation. This is thus the first demonstration of habitual responding on a heterogenous action sequencing task in rodents and all subsequent experiments in this study used this version of the task.

Unfortunately rats rapidly ceased responding under extinction conditions, producing very few complete sequences, which was not unexpected given the sequencing task uses a continuous reinforcement schedule (see Figure 1F noting five nose pokes were required per sequence). This led to floor effects for measuring sequencing behavior (such as timing or effects on initiation, execution, and terminal elements) and required the assessment of total nose pokes rather than sequences performed. The significant 3-way interaction on the repeated-measures ANOVA indicated that the flexible group showed outcome devaluation sensitivity on every hole (p<0.001), whereas the invariant group responded habitually on nose pokes 2, 4 and 5 (p’s>0.08) but showed outcome sensitivity on nose pokes 1 (p=0.02) and 3 (p=0.04). However, there was no significant difference in the frequency of nose pokes across holes 1-5 within either the valued or devalued session for the invariant group, indicating that rats did not perform the initiation, execution, or termination elements significantly more under either condition. They reduced responding across all holes, indicative of the sequence becoming chunked into a single motor plan that was no longer under goal-directed control. Although devaluation is often considered the ‘gold-standard’ test for detecting habits, the lack of whole sequences performed and limited scope for reliably detecting differences between experimental groups where smaller effect sizes were expected, prevented its use in subsequent experiments.

In a separate cohort of rats, we then measured hallmark traits of automaticity - increased speed and reduced variability. Acquisition of sequencing was observed over 15 sessions that were grouped into five blocks of three sessions (Figure 1G). Data from treatment-naïve rats (n=36) trained on the finalised version of the sequencing task (see Figure 1G), indicated that from the first to last block there was a significant reduction in correct nose poke duration (Block: F_(1,34)_= 18.12, p<0.001), which varied at each nose poke location (Block X Hole: F_2,82_=19.80, p<0.001; pairwise comparisons p’s<0.025; repeated-measures ANOVA) (Figure 1I). There was also a main effect of Hole (F_3,94_=35.31, p<0.001). By the last block, each action in the sequence became less variable as measured by a significant reduction in the coefficient of variance (CV=mean/standard deviation) from first to last block (Block: F_1,31_=88.02, p<0.001; Hole: F_4,124_=15.6, p<0.001; Block x Hole: F_3,96_=8.16, p<0.001; repeated-measures ANOVA). Post-hoc comparisons revealed nose poke duration was significantly less variable on the last block compared to the first block at every hole (p’s<0.001), indicative of refined and automated action sequencing. In the first block, a repeated-measures ANOVA found a main effect of Hole (F_3,88_=9.54, p<0.001) with post-hoc comparisons across holes indicating nose poke responses to holes 1, 2 and 5 were not significantly different (p’s>0.3), but those on holes 3 and 4 were faster (p’s<0.001), as indicated by the u-shaped curve in Figure 1I. By the end of acquisition, a ballistic response pattern had developed with responses speeding up across the sequence in the last block (Hole: F_2,72_=56.3, p<0.001). Post-hoc comparisons revealed responses on hole 1 took significantly longer than all other holes (p’s<0.001), then hole 2 responses took longer than holes 3, 4 and 5 (p’<0.009), then hole 3 responses were slower than hole 4 (p<0.001). Hole 4 responses were the fastest (p’s<0.001) with nose pokes in hole 5, the terminal action, significantly slower than mid-sequence responses at holes 3 and 4 (p’s<0.025), but faster than the first two responses at holes 1 and 2 (p’s<0.01). Here the rat anticipates and prepares the next motor chunk - reward retrieval. This response pattern, particularly the initiation and termination delays, are characteristic of motor sequence chunking (Sternberg et al., 1978; Abrahamse et al., 2013). Therefore, the sequential nose poke task leads to chunked action sequencing with features of both skill and habit formation as defined by rapid execution, invariant response pattern, evidence of sequence chunking and insensitivity to changes in outcome value (Balleine and Dezfouli, 2019).

### DMS-lesioning improved acquisition of action sequencing, while DLS-lesioning impaired efficient sequencing.

#### Initial training

**Figure 2:**
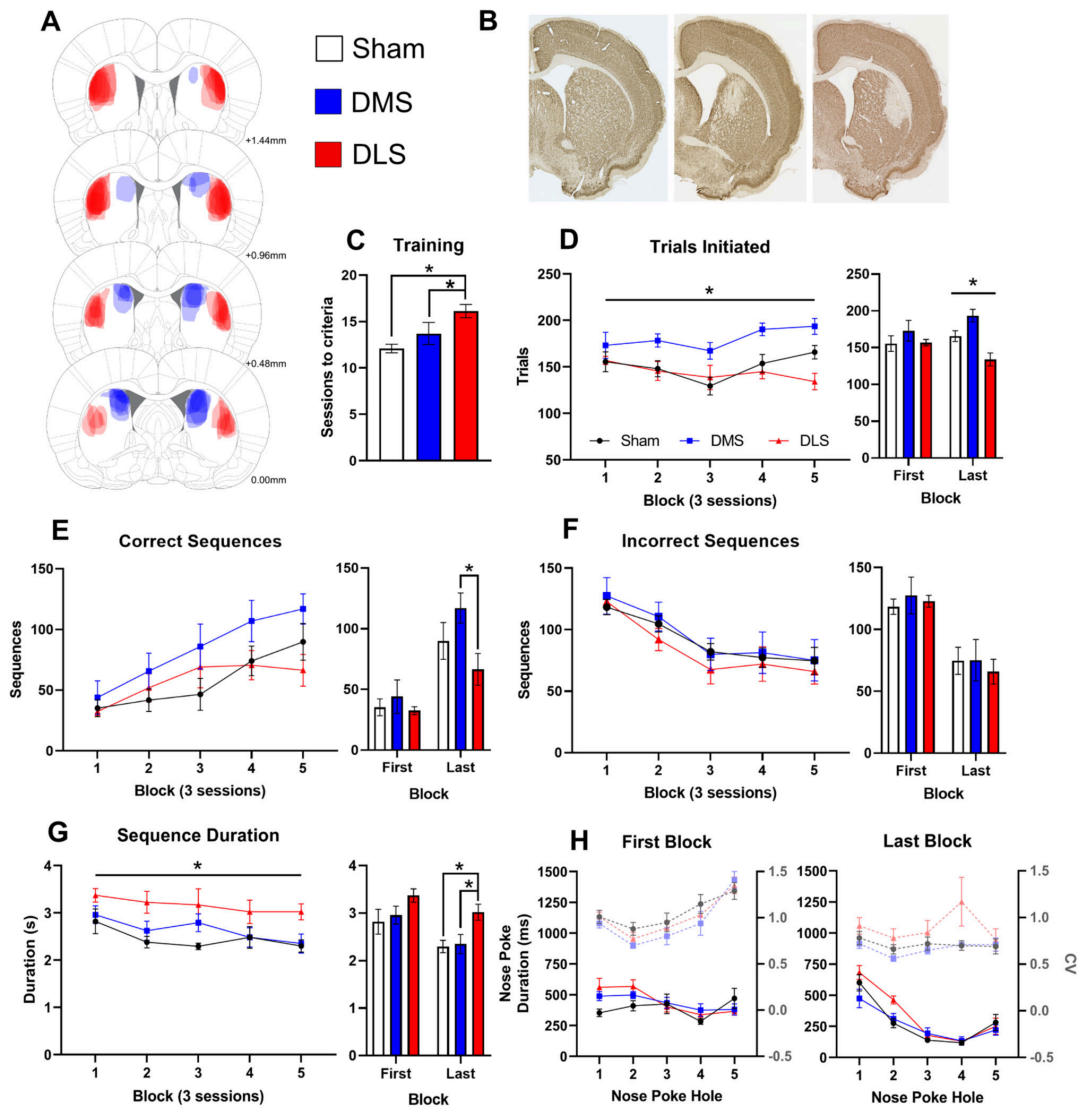
DMS-lesioning improved acquisition of action sequencing, while DLS-lesioning impaired efficient sequencing. (A) Rats received targeted bilateral lesions with extent illustrated for lesion groups; sham (open, n=11), DMS (blue, n=7), DLS (red, n=8). (B) Striatal sections showing NeuN staining in sham (left), DMS (middle) and DLS (right) lesioned rats. (C) DLS-lesioned rats required significantly more sessions to reach training criteria than sham or DMS-lesioned rats. (D) Left: When acquiring sequencing behavior, DMS-lesioned rats initiated significantly more trials than either DLS-lesioned or sham rats. Right: There was no significant difference between groups in the first block, however by the last block, DLS-lesioned rats started fewer trials and DMS-lesioned rats completed more trials than sham. (E) Left: Contrasting effects of lesions were also observed for the number of correct sequences. Right: DMS-lesioned rats completed nearly twice as many correct sequences than DLS-lesioned rats in the last block of acquisition. (F) Incorrect sequences decreased across acquisition, demonstrating all groups learned to avoid errors. (G) Left: Overall, DLS-lesioned rats took significantly longer to complete sequences than sham rats. Right: All groups completed sequences significantly faster from the first to last block of acquisition and in the final block DLS-lesioned rats took significantly longer to complete sequences than sham and DMS-lesioned rats. (H) Across acquisition, the variation and duration of nose pokes became faster and developed a ballistic response pattern. By the last block, DLS-lesioned rats paused significantly longer than DMS-lesioned rats on the first two actions of the sequence, but not the latter half of the sequence. Sham and DMS-lesioned rats had a significant reduction overall in variation from the first to last block, but DLS-lesioned rats did not. There was a significant interaction in DLS-lesioned rats as only variation on hole 5 reduced with training. Data shown as group mean ± S.E.M. *p<0.05.

To determine if the DMS and DLS work cooperatively or in opposition, subregion-specific loss of function was required throughout training and 15 sessions of sequence acquisition (Figure 2A, B). Lesions made via discrete fiber-sparing quinolinic acid infusions avoided any overlap between the DMS and DLS. Following recovery, rats were food restricted and trained on the sequencing task (see Figure 1C for schedule). Our a priori hypothesis was that we would observe divergence between DMS and DLS groups and hence direct comparisons were made. DLS-lesioned rats took significantly longer to reach training criteria (Figure 2C; Lesion: F_2,23_=7.80, p=0.003, univariate ANOVA) than DMS-lesioned (p=0.045) or sham treated rats (p=0.001). Rats then moved to sequence acquisition where only perfect 5-step sequences were rewarded.

#### Sequence acquisition

We compared performance measures during acquisition to quantify action sequence refinement, with a focus on changes between the first (sessions 1-3) and last blocks (sessions 12-15). Across the five blocks of acquisition, DMS-lesioned rats initiated more trials (Figure 2D; Lesion: F_2,23_=6.94, p=0.004, repeated-measures ANOVA) than either DLS-lesioned (p=0.002) or sham control (p=0.005) rats. The number of trials initiated was equivalent between groups in the first block (p>0.3). However, by the last block there were opposing effects detected between groups (Lesion: F_2,23_=11.59, p<0.001, repeated-measures ANOVA). Post-hoc comparisons revealed DLS-lesioned rats initiated significantly fewer trials than sham rats (p=0.09), while DMS-lesioned rats completed significantly more trials than sham rats (p=0.025); with a substantial difference between DMS and DLS groups (p<0.001). This acquired divergence between DMS and DLS lesioned rats demonstrated that DMS-lesions enhanced, while DLS-lesions impaired, initiation of action sequences. As sessions were time limited, performing more trials indicated greater speed and opportunity for reward, however these trials could have been either correct or incorrect.

The number of correct sequences increased for all groups across acquisition, indicating all groups were able to learn the five-step sequence. Opposing effects of striatal lesions were again also observed in the total number of correct sequences. There was no difference between groups on the first block, yet there was a clear divergence between DMS and DLS lesioned rats across acquisition (Figure 2E). Our a priori hypothesis was that DMS and DLS lesions would have opposing effects and a comparison between these lesion groups found that DMS-lesioned rats completed nearly twice as many correct sequences as DLS-lesioned rats at the end of acquisition (DMS = 117±12, DLS = 67±13; t_13_=2.79, p=0.015, t-test). Despite the dissociation between groups in both the number of sequences initiated and correct sequences, there was no difference in the number of incorrect sequences made by each group (Figure 2F; F_2,23_=0.16, ns, repeated-measures ANOVA). All groups showed a significant reduction in incorrect sequences from the first to last block (p’s<0.01) and these results indicated the differences observed between the groups were not due to general effects on learning. These results support a model of sequence learning where the DMS and DLS have opposing roles in the development of automated behaviors.

#### Sequence timing

We next investigated how striatal lesions influenced the timing of actions within sequences. Across sequence acquisition, sequence duration significantly reduced (Figure 2G; Block: F_4,92_=6.74, p<0.001, repeated-measures ANOVA), indicating increased sequencing efficiency with experience. This is important as faster execution is considered one of the hallmarks of skill learning and sequence chunking. Throughout acquisition, DLS-lesioned rats took significantly longer to execute complete sequences (Lesion: F_2,23_=4.59, p=0.021, repeated-measures ANOVA) than sham rats (p=0.007), with a trend towards impairment compared to DMS-lesioned rats (p=0.059). All groups completed sequences significantly faster from the first to last block of acquisition (sham, t_10_=2.33, p=0.042; DMS, t_6_=4.78, p=0.003; DLS, t_7_=2.83, p=0.026; t-tests). In the final block, DLS-lesioned rats took significantly longer to complete sequences (Lesion: F_2,23_=5.87, p=0.009, univariate ANOVA) than sham (p=0.004) and DMS-lesioned rats (p=0.013), supporting the conclusion that DLS lesions impaired the development of refined action sequencing.

As the task utilised five spatially heterogeneous responses, the timing of each action within the sequence was then compared across the initiation (hole 1), execution (holes 2-4) and terminal (hole 5) responses as well as the nose poke duration within each hole. Correct nose poke duration became faster across acquisition and developed the characteristic accelerating response pattern (Figure 2H; Block: F_4, 92_=19.57, p<0.001; Block X Hole: F_16,368_=9.07, p<0.001, repeated measures ANOVA). There were no significant differences between groups in the first block of acquisition (Figure 2H; Lesion: F_2,23_=0.67, ns, univariate ANOVA). However, by the last block, nose poke duration had stabilised to a ballistic response pattern and the variance in timing had reduced as the movement became stereotypical. To assess sequencing variation as a measure of automaticity, CV of nose poke duration on each hole from the first and last block was compared for each group (Figure 2H). Sham rats significantly reduced variation across training, especially for nose pokes 4 and 5 (Block: F_1,10_=23.43, p<0.001; Hole: F_4,40_=4.37, p=0.005; Block X Hole: F_4,40_=3.87, p=0.009; repeated-measures ANOVA). DMS-lesioned rats also significantly reduced variation across training with nose pokes in hole 5 showing the greatest reduction in variance (Block: F_1,6_=16.95, p=0.006; Hole: F_4,24_=16.46, p<0.001; Block X Hole: F_4,24_=9.77, p<0.001; repeated-measures ANOVA). Finally, in the DLS-lesioned rats there was no significant reduction in variance with training or across holes but there was a significant interaction as nose poke duration on hole 5 became less variable with training (Block: F_1,6_=1.24, ns; Hole: F_2,9_=2.42, ns; Block X Hole: F_4,24_=5.17, p=0.004; repeated-measures ANOVA). These results show that Sham and DMS-lesioned rats performed sequencing action with less variance after training, but DLS-lesioned rats did not, indicative of impaired automaticity.

On the last block, there was a main effect of Hole and the Hole X Lesion interaction approached statistical significance (Hole: F_2,49_=67.84, p<0.001; Hole X Lesion F_4,49_=2.51, p=0.051; repeated-measures ANOVA). Planned post-hoc comparisons found DLS-lesioned rats paused significantly longer than DMS-lesioned rats on the first two actions of the sequence (hole 1, t_13_=2.28, p=0.040 and hole 2, t_13_=2.92, p=0.012, t-test) but not the latter half of the sequence (p’s>0.7). These results demonstrated that while DLS-lesioned rats were capable of extremely fast nose poke responses (see hole 4) and therefore were not exhibiting general motor impairments, they were significantly delayed in initiating the sequence. These results suggest that the DLS is important for action selection and/or retrieval. However, once the sequence was engaged, its execution was not dependent on intact DLS function.

The inter-poke interval between correct nose pokes also speeded with training indicating improved efficiency. There was a u-shaped pattern across the curved wall, likely reflecting ambulation requirements but there was no effect of lesion or interactions with treatment groups (Hole: F_3, 45_=44.88, p<0.001; Lesion: F_2, 23_=1.04, ns; Hole X Lesion, p>0.05; repeated-measures ANOVA). There was also no effect of group on the latency from leaving the magazine to starting at hole 1, indicating all groups were equally as motivated to initiate sequences. There were also no significant changes in magazine nose poke duration or reward collection latency over acquisition or between groups suggesting training and lesions did not alter reward motivation.

Across numerous measures of performance, our results showed that DMS lesions accelerate the shift towards automatisation as demonstrated by increased accuracy, speed and reduced sequence duration variability across training. However, DLS lesions impaired the development of efficient action sequencing observed through the lack of improvement in accuracy, speed and variation across acquisition. Delayed sequence initiation but not execution or termination in DLS-lesioned rats, suggest that the DLS is important for loading the motor program, but once rats started responding the transition between elements was accurate and rapid, indicative of action sequence chunking. The role of cortical inputs may be critical in modulating this striatal balance (Daw et al., 2005; Lee et al., 2014; Peak et al., 2019). Cortical inputs to the striatum play an important role in both adaptive and habitual responding therefore we sought to determine whether subregions within the prefrontal cortex influence the acquisition of action sequencing. We hypothesised that cortical regions with inputs into the DLS would impair sequence acquisition, while those with inputs to the DMS may enhance acquisition.

### Lateral OFC but not medial OFC lesions impair sequencing

We first examined the role of the medial (mOFC) and lateral (lOFC) orbitofrontal cortex, which project to medial and lateral regions of the dorsal striatum, respectively. mOFC lesions lead to habitual responding via an inability to retrieve outcome value in outcome devaluation tests (Bradfield et al., 2015; Bradfield et al., 2018). In contrast, the lOFC is well known for its role in flexible responding in reversal learning, outcome prediction and sensitivity to devaluation (Gremel and Costa, 2013; Panayi and Killcross, 2014; Gremel et al., 2016; Izquierdo, 2017; Hervig et al., 2020; Turner and Parkes, 2020). Here we examined if either OFC region is required for the development of action sequencing.

#### Acquisition of sequencing

**Figure 3:**
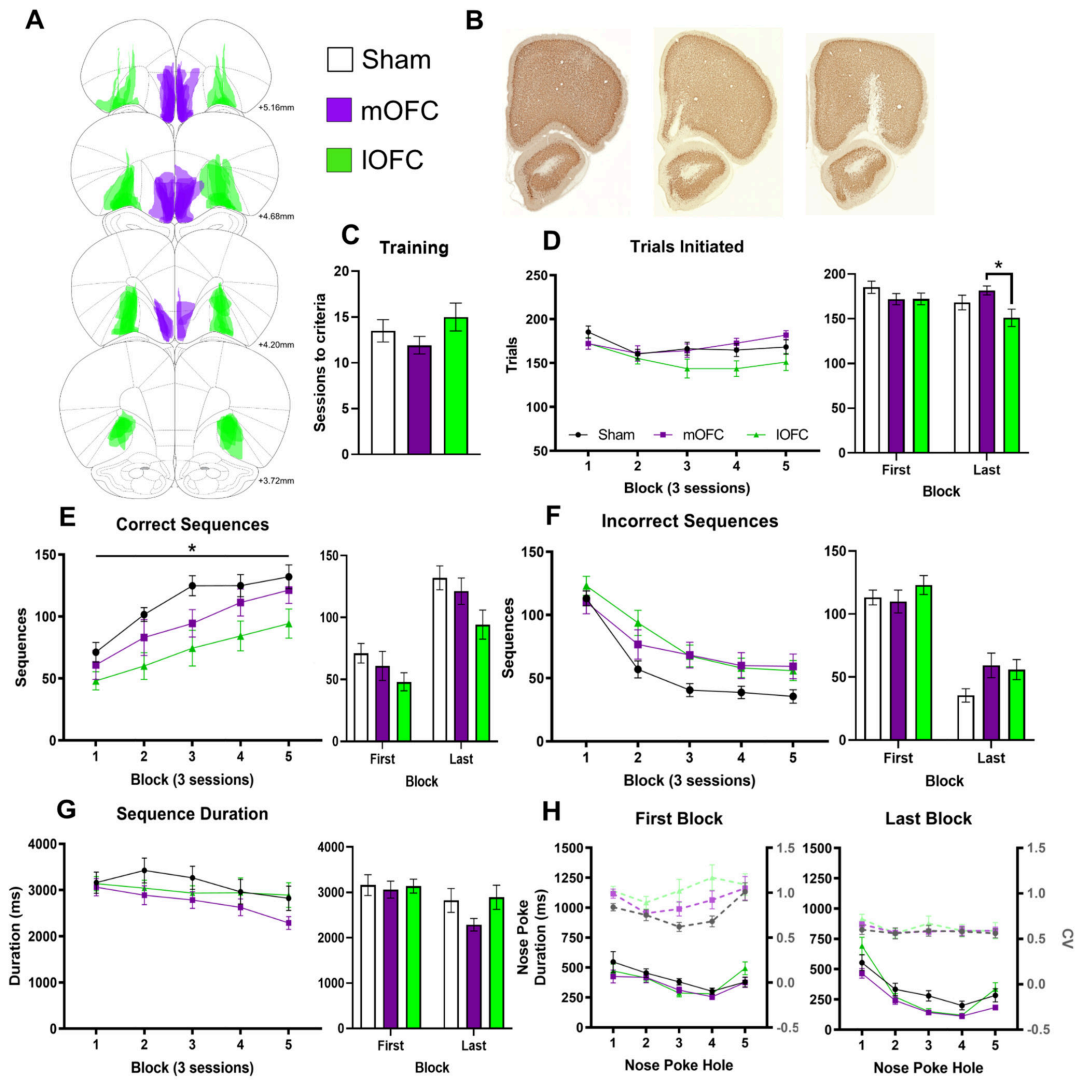
Lateral OFC but not medial OFC lesions impair sequencing. (A) Rats received targeted bilateral lesions as shown for sham (open, n=10), mOFC (purple, n=8) or lOFC (green, n=12). (B) Sections showing NeuN staining for sham (left), mOFC (middle) and lOFC (right) lesion groups. (C) Sessions to reach training criteria was not different between groups. (D) The number of trials initiated was not different in the first block, but signficantly reduced in lOFC- compared to mOFC-lesioned rats after acquisition. (E) lOFC-lesioned rats producing significantly fewer correct sequences than sham rats across acquisition. (F) All rats significantly reduced incorrect sequences over acquisition. During early acquisition, lOFC-lesioned rats continued to make more incorrect sequences in block 2 and both lesion groups made more errors in block 3 compared to the sham group. (G) There was a trend for reduced sequence execution time across acquisition, however only the mOFC-lesioned group significantly reduced sequence duration from the first to last block. (H) At the end of acquisition, nose poke duration was faster in lesioned rats than sham controls in the middle of the sequence, however lOFC-lesioned rats were slower at terminating the sequences compared to mOFC-lesioned rats. All groups had reduced variation in nose poke duration at the end of acquisition. Data shown as group mean ± S.E.M. *p<0.05.

Using the same procedure, we determined if the mOFC and lOFC were required for action sequencing (Figure 3A, B). There was no effect of lesions on the number of sessions required during training (Figure 3C) but across sequence acquisition, there was a significant interaction between groups (Figure 3D; Lesion X Block: F_8, 180_=2.72, p=0.024; repeated-measures ANOVA). There was no difference between groups in the first block, but by the end of acquisition the lOFC-lesioned rats initiated fewer trials than mOFC-lesioned rats (Lesion: F_2,27_=4.49, p=0.021; univariate ANOVA with post-hoc comparison p=0.006). lOFC-lesioned rats were also the only group to show a significant *reduction* in trials completed from the first to last block (t_9_=3.17, p=0.011; paired t-test). There was a main effect of Lesion on the number of correct sequences completed (Figure 3E; Lesion: F_2, 27_=3.55, p=0.043; repeated- measures ANOVA) with lOFC-lesioned rats producing significantly fewer correct sequences than sham treated rats (p=0.014, post-hoc comparison) throughout acquisition. There was also a significant interaction for the number of incorrect sequences produced across acquisition (Figure 3F; Block: F_2, 67_=101.3, p<0.001; Lesion X Block: F_5, 67_=2.59, p=0.034; repeated-measures ANOVA). This interaction was most evident in the early blocks with more errors from lOFC-lesioned rats compared to sham rats in block 2 (p=0.026; post-hoc comparison). Together, these results show that lOFC-lesioned rats were producing fewer correct and more incorrect sequences, suggesting they were impaired in learning from negative feedback. This interaction also indicated mOFC-lesioned rats made more incorrect sequences in block 3 (compared to sham: mOFC p=0.042, lOFC p=0.055; post-hoc comparison) but did not have any other deficits, suggesting this was a subtle impairment.

#### Sequence timing

There was a significant reduction in total sequence duration across acquisition without any significant different between groups (Figure 3G; Lesion: F_1,27_=1.39, ns; Block: F_3,77_=11.11, p<0.001; Lesion X Block: F_8,108_=1.15, ns; repeated-measures ANOVA). Rats became significantly faster at executing correct nose pokes from the first to last block (Block: F_1,27_=26.28, p<0.001; Lesion: F_2,27_=3.04, p=0.064; repeated-measures ANOVA) with a significant Block X Hole interaction (Figure 3H; F_4, 108_=22.33, p<0.001) as response times shifted to a ballistic response pattern with training. There was also a significant Lesion X Hole interaction (F_5,64_ =2.68, p=0.032) with both lesion groups making faster responses in the middle of the sequence than sham rats (hole 3 p’s<0.003; post-hoc comparisons), yet lOFC-lesioned rats were significantly delayed on the terminal action in the sequences compared to mOFC-lesioned rats (hole 5, p=0.011). To examine automaticity, the variance of each nose poke response was compared across acquisition (Lesion: F_2,27_=3.26, ns; Block: F_1,27_=40.50, p<0.001; Hole: F_4,108_=6.65, p<0.001; Lesion X Block: F_2,27_=1.34, ns; Lesion X Block X Hole: F_6,75_=1.48, ns; repeated-measures ANOVA). There was a significant reduction in variance across training with a trend towards overall higher variation in lOFC-lesioned rats, but no significant interaction between Block and Lesion, indicating all groups showed a reduction in variation with training. There was no significant change in the duration of time spent in the magazine or latency to collect the reward. There was a trend for lOFC-lesioned rats to approach the magazine faster by block 5 while mOFC and sham rats were slower (Lesion: F_2,27_=0.97, ns: Block: F_1,27_=3.42, p=0.075: Block X Lesion: F_2,27_=3.20, p=0.057; repeated-measures ANOVA). The latency from leaving the magazine to nose poke 1 reduced from block 1 to 5, but did not differ by groups (Lesion: F_2,27_=0.25, ns: Block: F_1,27_=6.88, p=0.014: Block X Lesion: F_2,27_=2.13, ns; repeated-measures ANOVA). The inter-poke intervals were also not significantly different for lesioned rats overall (Lesion: F_2,27_=1.21, ns: Block: F_1,27_=55.0, p<0.001: Block X Lesion: F_2,27_=4.34, p=0.023; Block X Lesion X Hole: F_6,51_=2.36, p=0.068; repeated-measures ANOVA), although lOFC-lesioned rats were slower than sham (p=0.029, post-hoc comparison) on the first block but all groups were equal by the final block.

In summary, lOFC-lesioned rats were impaired across many measures of action sequence acquisition. While they performed as well as sham rats in the first block, they did not adapt efficiently to the requirement to only produce invariant sequences. This was evidenced by the more gradual reduction in incorrect sequences, consistently fewer correct sequences and start/stop delays observed when initiating and terminating sequences (despite unimpaired mid-sequence execution). Given shared impairments in initiating sequencing, the lOFC to DLS projection may be important for loading motor sequences. This is in contrast to mOFC-lesions, which reduced their sequence duration across acquisition, but also produced more incorrect responses during acquisition, unlike the enhancing effects of DMS lesions.

### Prelimbic and infralimbic cortex lesions do not alter sequence acquisition

To further understand the role of the medial prefrontal cortex, we next examined the effects of excitotoxic lesions of the prelimbic (PrL) and infralimbic (IL) cortex. These regions are associated with goal-directed and habitual behavior respectively, with the PrL having strong inputs to the DMS and the IL into the ventral striatum (Coutureau and Killcross, 2003; Mailly et al., 2013; Hart et al., 2014; Heilbronner et al., 2016).

#### Acquisition of sequencing

**Figure 4:**
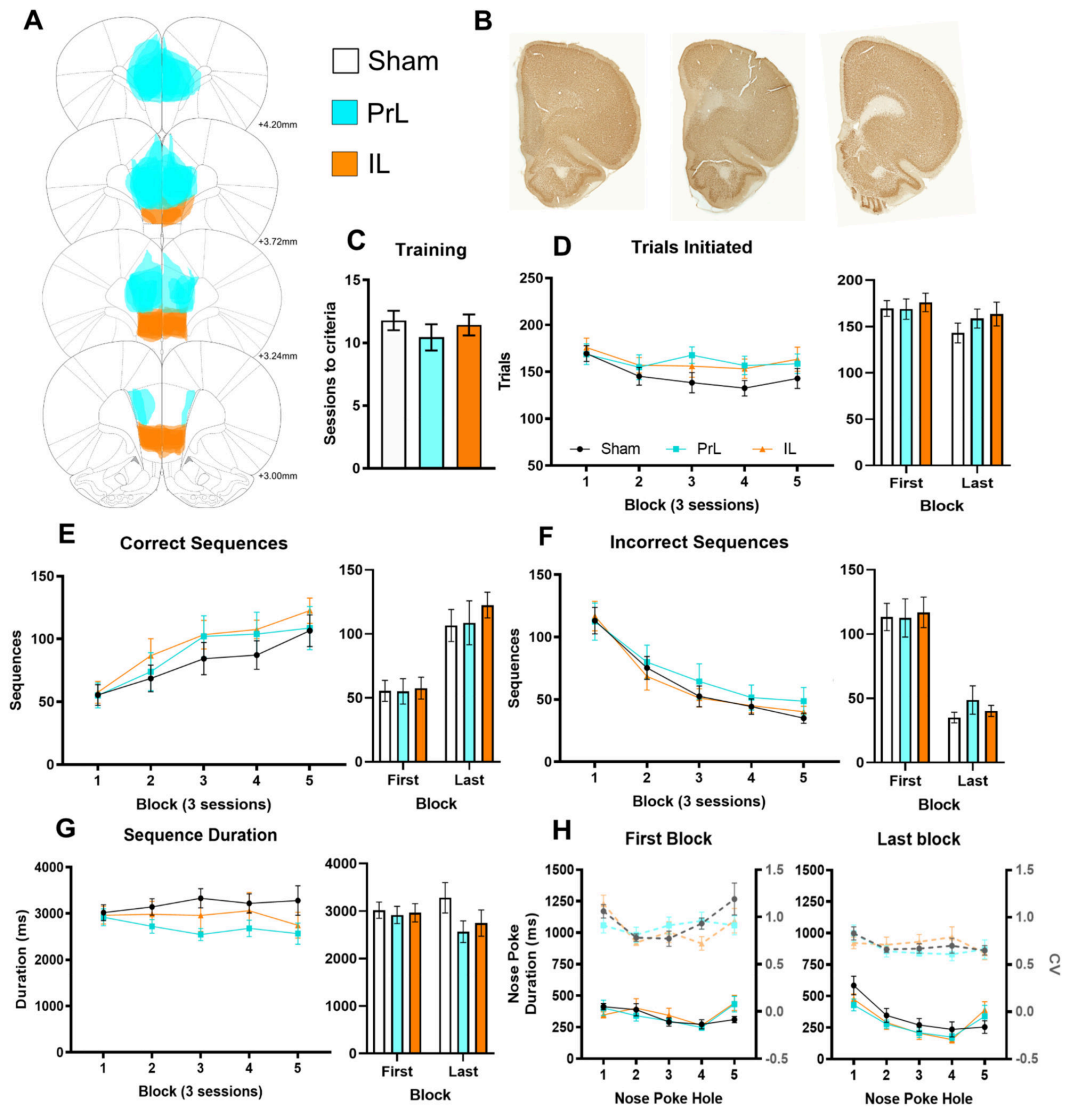
Prelimbic and infralimbic cortex lesions do not alter sequence acquisition. (A) Rats received targeted bilateral lesions as shown for sham (open, n=9), PrL (cyan, n=9) or IL (orange, n=7). (B) Sections showing NeuN staining in sham (left), PrL (middle) and IL (right) lesion groups. (C) The number of trials initiated was not different between groups. (D) The number of correct sequences significantly increased without an effect of lesion. (E) Incorrect sequences significantly decreased, and this was also not different between groups. (F) Rats became significantly faster at executing the sequence with training with no significant differences between groups. (G) Total sequence duration reduced across the acquisition period but was not different between groups. (H) Nose poke duration shifted to the characteristic accelerating pattern with no effect of PrL or IL lesion and all groups had reduced variation at the end of training. Data shown as group mean ± S.E.M.

Identical procedures were implemented in PrL and IL lesioned rats (Figure 4A, B). All groups reached criteria before moving onto the sequence acquisition (Figure 4C). The number of correct sequences significantly increased across acquisition (Figure 4E; Block: F_2, 51_=26.57, p<0.001; repeated-measures ANOVA) and incorrect sequences significantly decreased (Figure 4F; Block: F_2, 41_=58.93, p<0.001; repeated-measures ANOVA) with no effect of treatment or interactions on trials initiated (Figure 4D) or the number correct or incorrect sequences (Figure 4E, F).

#### Sequence timing

While there was a main effect of Block (Figure 4G; F_3, 64_=2.95, p=0.041; repeated-easures ANOVA) on total sequence duration where rats became significantly faster at executing the sequence with training there was no significant difference between lesion groups for correct nose poke duration across sequence or magazine, inter-poke intervals between holes, or interval from hole 5 to the magazine. Nose poke duration did reduce from first to last block across all lesion groups and a significant interaction identified the ballistic-like response pattern developing with training (Figure 4H; Block: F_1, 22_=7.61, p=0.011; Block X Hole: F_4, 2_=8.64, p<0.001; repeated-measures ANOVA). Variability also reduced but did not differ between groups (Figure 4H; Block: F_1, 22_=35.20, p<0.001; Hole: F_4, 56_=7.13, p<0.001; Lesion: F_2 22_=0.16, ns; Block X Hole: F_4, 6_=4.37, p=0.008; repeated-measures ANOVA). These results indicated that the PrL and IL cortex were not critical for the acquisition of action sequencing.

## Discussion

We found heterogenous action sequences were under habitual control when the parameters of this task promoted automaticity and provide the first direct causal evidence that the DMS and DLS have opposing roles on the acquisition of sequencing. The finding of striatal opposition is consistent with studies showing concurrent activity within the DMS and DLS across numerous tasks, and that disengagement of the DMS predicts skill learning by allowing the DLS to take control (Thorn and Graybiel, 2014; Kupferschmidt et al., 2017). The finding is also consistent with the DMS gating habit formation, as although the DLS is active during early learning it only gains control when DMS activity subsides (Thorn et al., 2010). While the lOFC was required for action sequencing, surprisingly lesions to medial prefrontal cortical subregions (mOFC, PrL and IL) neither impaired nor enhanced acquisition of action sequencing. Together, these results demonstrate that reduced DMS activity facilitates the acquisition of DLS-dependent behavior, but the source of arbitration between these parallel corticostriatal loops is independent of medial prefrontal inputs to the dorsal striatum.

## Opposing role of the dorsal striatum in the acquisition of action sequences

Previous studies have shown habitual responding can be acquired despite DMS lesions (Hilario et al., 2012; Gremel and Costa, 2013), suggesting that a DMS-dependent goal-directed acquisition phase is not required for habit development. We provide evidence for this hypothesis by demonstrating that DMS-lesioned rats show *enhanced* acquisition of this habit-like response pattern. These results not only indicated that DMS-dependent learning was not critical for efficient task acquisition, but that the DMS may hamper the development of automatic action sequencing. Studies of flexible behavior have found DMS-lesions impair performance as anticipated, given the role of the DMS in goal-directed behaviors. However, there is some evidence that loss of DLS function can *enhance* learning in rodents, suggesting a competitive influence of DLS functions on DMS-dependent behaviors (Moussa et al., 2011; Bradfield and Balleine, 2013; Bergstrom et al., 2018). We demonstrate that the converse is true for automatisation of actions, indicating this competitive relationship is bidirectional.

## Habits, skills and automaticity

We capitalised on a task that is dependent on reduced behavioral variation (rather than overtraining) to examine the neural underpinnings of automatisation, reflecting shared features of habits and skills. How the similarities and differences between habits and skills can be consolidated has been a question of growing interest that remains largely unanswered (Ashby et al., 2010; Graybiel and Grafton, 2015; Robbins and Costa, 2017; Hardwick et al., 2019). Automaticity is commonly measured in skill learning using tasks such as rotarod (Yin et al., 2009; Kupferschmidt et al., 2017) and action sequencing paradigms, including fixed ratio or shorter two- or four-step sequencing tasks (e.g. L-R lever press) (Yin et al., 2005b; Yin, 2009, 2010; Wassum et al., 2012; Cui et al., 2013; Jin et al., 2014; Tecuapetla et al., 2016; Geddes et al., 2018; Garr and Delamater, 2019). However, to our knowledge, models of skill and habit formation have not been tested hitherto in rodent operant paradigms requiring more than two different response elements. We found that DLS-lesions specifically affected initiation rather than execution elements, consistent with the suggestion that DLS activity is important when starting and stopping motor sequences, rather than the mid-sequence actions, which is evident in task bracketing patterns (Sternberg et al., 1978; Jin and Costa, 2010). Instead of identifying the specific motor actions that will be performed, DLS activity may be important for bracketing groups of familiar motor actions as a chunk (Smith and Graybiel, 2013). Our results support this suggestion as DLS-lesioned rats had no deficits in performing the five actions in the correct order and displayed a ballistic response pattern synonymous with chunking but were impaired when starting the sequence. Plausibly, the DLS is important for retrieving and initiating rehearsed behavioral patterns, promoting their rapid, stimulus-driven and refined expression, with important implications for the role of the DLS in automaticity, habits, and skill formation.

## Cortical contributions to skilled action sequencing

Cortical inputs may play a critical role in habit and skill development but less is known about how they operate across transitions and in action sequences (Killcross and Coutureau, 2003; Ostlund et al., 2009; Smith and Graybiel, 2013; Bergstrom et al., 2018; Bradfield et al., 2018; Turner and Parkes, 2020). A correlational link between cortical disengagement and skill refinement has been observed using neuroimaging in humans (Bassett et al., 2015) and neuronal recordings in rodents (Kupferschmidt et al., 2017). Previous research has associated PrL with goal-directed actions and the IL with habits. Using a lesioning approach, our results provide the first evidence that these regions are not required to learn and perform heterogenous action sequences.

The PrL cortex is important for early stages of goal-directed learning but not for habit formation (Corbit and Balleine, 2003; Coutureau and Killcross, 2003; Hart et al., 2018b), consistent with the lack of effect in this study. The fact that PrL-lesions did not *enhance* sequencing indicates that the PrL inputs to the DMS are not solely responsible for maintaining DMS functions or goal-directed interference on this task. This independence of functions suggests the switch in control within the dorsal striatum is not driven by the PrL cortex.

Lesioning the IL did not impair sequence acquisition as predicted from devaluation studies showing IL-lesions result in goal-directed responding (Coutureau and Killcross, 2003). Shipman et al. (2018) suggested that control shifts from the PrL to IL with experience but prior to habit formation, highlighting a role in the transition of control. Further, Smith and Graybiel (2013) proposed that the IL and DLS operate together to establish habits, however there were differences between the electrophysiological signatures of DLS and IL in habits (e.g., after devaluation), and there are no direct IL-DLS projections, suggesting they have independent roles in habitual responding. In addition, IL activity does not reflect the habitual nature of individual decisions, indicating it is not arbitrating between goal-directed and habitual strategies but instead reflects overall response tendencies or states (Smith and Graybiel, 2013). Haddon and Killcross (2011) found that the IL plays a role when goal-directed and habitual associations are in competition. This finding is then consistent with our lack of effect of IL lesions on sequence acquisition when there was only minimal competition.

While the effects of lOFC lesions were largely consistent with deficits in DLS-lesioned rats, two key differences emerged. lOFC lesioned rats were relatively slower to terminate sequences and had higher rates of incorrect responses without impairments in automaticity as measured by response variance. The terminal delay in our study, as well as the delayed reward collection latency reported in Hervig et al. (2020), may be due to the lOFC’s role in predicting outcomes based on Pavlovian cues, reward delivery being cued (Ostlund and Balleine, 2007; Panayi and Killcross, 2014). This is important given the lOFC is implicated in perseverative and compulsive behaviors, lacking appropriate termination (Chudasama and Robbins, 2003; Burguiere et al., 2013). The lOFC has also been implicated in credit assignment, of likely importance when chaining a series of actions for which only the final element is rewarded (Noonan et al., 2017). Prior studies have found that large lOFC lesions produced similar effects to those seen in DMS-lesioned animals performing under both random ratio and random interval schedules (Gremel and Costa, 2013). The lack of devaluation sensitivity in both schedules following lOFC loss of function was suggested to indicate its role in conveying action-value information. Our results support this notion as an impairment in learning rather than in increase in habit formation or automaticity, given they made more incorrect responses and performed fewer sequences. Future studies should confirm if lOFC to DLS projections are critical for action sequencing and isolate the lOFC deficits linked to this specific pathway.

Overall, the cortical effects (or lack thereof) described here are problematic for the popular model of top-down control applied by cortical regions over subcortical structures. This may simply not apply in the same way to behaviors that dominate motor rather than cognitive cortico-striatal loops. This lack of effect is significant in the context of understanding where arbitration of striatal control originates and highlights the importance of considering tasks that optimise automatic, habitual actions to understand cortico-striatal function (Lee et al., 2014). Perhaps when there is little or no need for goal-directed control, there is also little need for medial prefrontal cortical input. However, no evidence of enhanced acquisition (as following DMS lesions) was observed, possibly due to the redundancy afforded by multiple cortical projections to the DMS.

## Conclusions

These findings provide strong evidence for competition between DMS and DLS functions in the development of behavioral automatisation. We found medial prefrontal subregions were largely unnecessary for sequence acquisition, however lesions to the lOFC impaired action sequencing. Developing an innovative spatial heterogeneous action sequencing task, we were able to isolate initiation, execution and termination specific deficits. These results provide empirical support for a model where DMS activity limits the formation of automated behavior, emphasising its role in the acquisition of skills and habits.

## Figures and Tables

**Table 1:** Summary of training stages and criteria to move to the next stage.

Stage	Summary	Criteria	Av. Sessions
**Stage 1**	Habituation to chamber	100 pellets x 1 session	1
**Stage 2**	Start nose poking 5 holes	>15 sequences x 1 session	7
**Stage 3**	Cued sequence – must NP	>50 sequences x 1 session	1
**Stage 4**	No cues	>50 sequences x 1 session	3
**Stage 5**	Incorrect = Time Out	Final stage	15

**Table 2:** Behavioral measures used to quantify action sequencing.

Trials	Total number of trials initiated
**Correct**	Number of completed sequences
**Incorrect**	Number of incorrect sequences
**Sequence Duration**	NP entry at NP1 to exit on NP5
**NP Duration**	Time from entry to exit of correct nose poke
**Inter-Poke Interval (IPI)**	Time from exit of previous NP to entry of next NP
**Initiation Latency**	Time from exit magazine to entry NP1 of next trial
**Reward Latency**	Time from exit NP5 to magazine entry when correct

**Table 3:** Co-ordinates and volumes used for pre-training lesion infusions of quinolinic acid. DMS: dorsomedial striatum; DLS: dorsolateral striatum; PrL: prelimbic cortex; IL: infralimbic cortex; mOFC: medial orbitofrontal cortex; lOFC: lateral orbitofrontal cortex; ant: anterior; post: posterior.

Region	AP	ML	DV	Volume (ul)
**DMS**	-0.4	+2.2	-4.5 (skull)	0.3
**DLS**	+0.7	+3.6	-5.0 (skull)	0.3
**PrL anterior**	+3.5	+0.7	-2.5 (dura)	0.3
**PrL posterior**	+2.8	+0.7	-2.8 (dura)	0.3
**IL anterior**	+2.9	+0.7	-4.0 (dura)	0.2
**IL posterior**	+2.5	+0.7	-4.0 (dura)	0.2
**mOFC**	+4.0	+0.6	-3.3 (dura)	0.3
**lOFC**	+3.5	+2.5	-3.6 (dura)	0.3

## Significance statement

We provide the most direct evidence to date that the dorsomedial and dorsolateral striatum compete for control in the acquisition of habitual action sequences. The dorsolateral striatum was critical for sequencing behavior, but loss of dorsomedial striatum function enhanced acquisition. In addition, we found that the medial prefrontal cortex was not required for the formation of automated actions. Using a task that optimises habitual responding, we demonstrate that the arbitration of dorsomedial and dorsolateral control is not modulated by medial prefrontal cortical activity. However, we find evidence for the role of the lateral orbitofrontal cortex in action sequencing. These results have implications for our understanding of how habits and skills form.
